# A Dual Face of APE1 in the Maintenance of Genetic Stability in Monocytes: An Overview of the Current Status and Future Perspectives

**DOI:** 10.3390/genes11060643

**Published:** 2020-06-11

**Authors:** Gabriela Betlej, Ewelina Bator, Antoni Pyrkosz, Aleksandra Kwiatkowska

**Affiliations:** 1 Institute of Physical Culture Studies, College of Medical Sciences, University of Rzeszow, 35-959 Rzeszow, Poland; ebator@ur.edu.pl (E.B.); akwiatkowska@ur.edu.pl (A.K.); 2 Medical College, University of Rzeszow, 35-959 Rzeszow, Poland; antoni.pyrkosz@gmail.com

**Keywords:** monocytes, APE1, genetic stability, tissue homeostasis, chromosomal instability, TNFα

## Abstract

Monocytes, which play a crucial role in the immune system, are characterized by an enormous sensitivity to oxidative stress. As they lack four key proteins responsible for DNA damage response (DDR) pathways, they are especially prone to reactive oxygen species (ROS) exposure leading to oxidative DNA lesions and, consequently, ROS-driven apoptosis. Although such a phenomenon is of important biological significance in the regulation of monocyte/macrophage/dendritic cells’ balance, it also a challenge for monocytic mechanisms that have to provide and maintain genetic stability of its own DNA. Interestingly, apurinic/apyrimidinic endonuclease 1 (APE1), which is one of the key proteins in two DDR mechanisms, base excision repair (BER) and non-homologous end joining (NHEJ) pathways, operates in monocytic cells, although both BER and NHEJ are impaired in these cells. Thus, on the one hand, APE1 endonucleolytic activity leads to enhanced levels of both single- and double-strand DNA breaks (SSDs and DSBs, respectively) in monocytic DNA that remain unrepaired because of the impaired BER and NHEJ. On the other hand, there is some experimental evidence suggesting that APE1 is a crucial player in monocytic genome maintenance and stability through different molecular mechanisms, including induction of cytoprotective and antioxidant genes. Here, the dual face of APE1 is discussed.

## 1. Introduction

Monocytes (MOs) are mononuclear phagocytic cells that are mostly produced in bone marrow and released into the bloodstream. In response to chemokines and cytokines, they are able to cross the walls of blood vessels, migrate to tissue, and differentiate into either dendritic cells (DCs) or macrophages (MAs). They are also called ‘the first line of defense’ because they take part in defending the host against infections, remove the infection agents by gobbling up the invading pathogens, and present antigens to lymphocytes in the immune resistance process. Monocytes are also involved in regenerating and maintaining the tissue homeostasis. They are engaged in both pro- and anti-inflammatory processes because of their ability to produce a wide range of cytokines that promote or inhibit inflammation, respectively.

Aberration in tissue regeneration and a prolonged immune response are undesirable and observed in diseases, such as fibrosis. Furthermore, mechanisms that instruct monocytes to adopt such properties are still unclear and are still being studied intensively. However, a deeper understanding of the molecular basis of some diseases might be pivotal for the development of therapy interventions.

## 2. Subsets of Monocytes and Their Main Functions

Monocytes are endowed with extremely high plasticity that manifests in their numerous functions and diversity. Although monocyte subsets should rather be considered a continuum [1], the simplifying view assumes that human monocytes are divided into three main subpopulations, based on their surface marker expression, such as CD14 and CD16. Amongst them, there are CD14^++^CD16^-^ (classical), CD14^+^CD16^++^ (non-classical), and CD14^++^CD16^+^ (intermediate) monocytes. Each of these types exhibit distinct properties. For example, classical MO are more efficient than others in reactive oxygen species (ROS) production and fungal infection reduction. After infection or injury, CD14^++^CD16^-^ monocytes arrive to tissue and produce a distinct set of chemokines that activate other immune cells. Non-classical monocytes, which are engaged in patrolling, are able to adhere and migrate along the lumen of the vessel. Moreover, they are characterized by a higher expression of chemokine receptors that are crucial for MO migration to damaged or inflamed tissues. Intermediate MOs play a key role in antigen presentation, and the secretion of cytokines or proinflammatory interleukins, such as: tumor necrosis factor α (TNFα), interleukin-1β (IL-1β), and interleukin-6 (IL-6) [2]. Moreover, they are potentially involved in the antitumor response [3,4] and may inhibit tumor cell growth through increases of proinflammatory cytokines and reactive nitrogen levels [5].

Monocytes play an important role in host defense. However, they also maintain the tissue homeostasis. Swirski and co-workers identified a monocyte reservoir in the spleen, which after ischemic myocardial injury accumulated in the injured tissue and was involved in wound healing [6]. Furthermore, MO are able to clear apoptotic or senescent cells without inducing immune responses or inflammation in the process called efferocytosis, which is another mechanism responsible for maintaining tissue homeostasis. Senescent cells are characterized by a specific secretory phenotype that may influence neighboring cells and its long-term accumulation disturbs proper tissue structure and function. However, efferocytosis is a challenge for monocyte cells over a relatively short period, especially in the context of large amounts of metabolic cargo that have to be processed. Although there is a number of mechanisms that prevent cells from accumulating potentially toxic cellular contents, defects in efferocytosis are considered to contribute to the pathogenesis of various diseases [7,8,9]. It is widely accepted that MOs play distinct roles in tissue repair; however, mechanisms that determine monocyte properties, such as anti- or proinflammatory fibrotic or regeneration ones, remain unclear and are intensively studied [10].

Nevertheless, in some cases, high counts or enhanced activity of MOs can be detrimental. For example, such a phenomenon is observed in fibrosis diseases, which are a consequence of tissue repair aberrations. This group of illnesses is associated with pathological gradual increases in organs’ remodeling, leading to the loss of their functions. Scott and colleagues observed that increased levels of MOs was associated with a shorter survival time and poor diagnosis. This research group also determined a single threshold value of monocytes that was associated with the high mortality of patients. Moreover, they proposed that this value may be useful to identify high-risk patients with an idiopathic pulmonary fibrosis [11]. It is supposed that the monocyte level may be a useful biomarker of other illnesses, such as coronary heart disease [12], sepsis-2 and sepsis-3 [13], amyotrophic lateral sclerosis [14], and many more. Here, the phase of tissue repair should be highly controlled and resolves quickly to restore normal tissue architecture [10]. This is why a highlight on wound healing and controlling monocytes’ function and lifespan might be key for understanding the molecular details of some diseases and to be exploited therapeutically.

## 3. Monocytes Are Impaired in DNA Repair Mechanisms and Accumulate DNA Strand Breaks

ROS have been shown to play an important role in the regulation of the MO level. As was mentioned above, monocytes increase the level of ROS and this mechanism, called “respiratory burst”, is pivotal to fight against pathogens and the removal of dead cells. However, an increased level of reactive oxygen species makes MO particularly vulnerable to oxidative damage. Paradoxically, it was shown that monocytes are impaired in DNA repair pathways, such as BER (base excision repair) and NHEJ (non-homologous end joining), which leads to an accumulation of SSB or DSB [15].

BER is the major mechanism, whose role is to correct base lesions, including oxidative modifications. This repair pathway is initiated by a glycosylase-dependent recognition and removal of a damaged base from the DNA strand that results in the creation of an apurinic/apyrimidinic (AP) site. In the next step, APE1, which has the capacity to cleave phosphodiester bonds in the double helix from the AP site, generates a nucleotide gap and the 3’OH free end [16]. The gap formation is re-ligated in the further steps of the BER and/or NHEJ pathways, which are executed by other proteins, such as X-ray repair cross-complementing protein 1 (XRCC1), DNA ligase IIIα (Lig IIIα), poly[ADP-ribose] polymerase 1(PARP-1), and DNA-dependent protein kinase, catalytic subunit (DNA-PKcs). XRCC1, Lig IIIα, and PARP-1 are also key players in SSB repair. These four proteins are thus crucial to successfully end the DDR process, but none of them are synthesized in monocytes. In contrast, their expression is activated in monocyte-derived macrophages and DCs. In this particular mechanism, APE1 significantly contributes to DNA damage in monocytes, as its endonucleolytic activity leads to the accumulation of SSBs and—due to the overlapping different DDR mechanisms—DSBs as well. This in turn strongly destabilizes the monocytic genome’s integrity and ultimately leads to cell death. Indeed, nonrepaired DSBs trigger the apoptotic pathway through the activation of ataxia telangiectasia mutated (ATM), ataxia telangiectasia, and Rad3 related (ATR), cell cycle checkpoint kinase 1 (Chk1), cell cycle checkpoint kinase 2 (Chk2), and tumor protein 53 (p53) [15]. Similar results were demonstrated previously by another research group that analyzed monocyte responses after exposure to methylating agents and the anticancer drug temozolomide (TMZ) [17].

A comprehensive study showed that monocytes treated with TMZ accumulated SSBs and DSBs, which, in turn, promoted apoptosis by activating the ATM-Chk2 and ATR-Chk1 pathways as well as a p53 level increase and activation through phosphorylation. Both ATR-Chk1 and ATM-Chk2 mechanisms, which are part of DDR (DNA damage response), are activated in response to SSBs and DSBs, leading to cell cycle arrest. Additionally, the involvement of caspases was also observed [18]. The high sensitivity of monocytes to TMZ probably depends on dysregulating expression and/or activity of the O6-methylguanine-DNA methyltransferase (MGMT), which is an important monocytic DNA repair protein [15]. The role of MGMT has also been documented during IL-4 and GM-CSF-dependent maturation of human monocytes into DCs [17].

Taken together, monocytes are DNA repair-incompetent cells and thus highly sensitive to agents, leading to DNA damage. From such a point of view, APE1, through its endonucleolytic activity that is not further processed by the orchestrated action of other BER and/or NHEJ proteins, acts as a factor destabilizing monocyte genome integrity.

On the other hand, APE1, through its involvement in other different molecular/biochemical pathways, seems to execute, directly or indirectly, a positive effect on monocyte genome stability maintenance. Some examples of such mechanisms with an emphasis on the putative role of APE1 in the maintenance of genome integrity and stability are presented and discussed below.

## 4. APE1 Gene and Its Protein Product

The human gene encoding APE1 has 3 kb and is located on chromosome 14 (14q 11.2–12) [19,20]. Its expression is observed in all cell types [21]. In turn, the protein product of APE1 consists of 318 amino acids and its mass is 35.6 kDa [19,20]. APE1 is the primary cellular endonuclease that plays a critical role in the BER mechanism. However, APE1 is also a component of other repair pathways [22]. As it was mentioned above, AP endonuclease, due to its C-terminal domain activity, is engaged in creating nicks in the DNA strand at the AP site, which is required in the DNA repair. Nevertheless, APE1 not only significantly contributes to DNA repair mechanisms but also has other activities that, in turn, are associated with the N-terminal domain and act independently from its endonuclease activity. The N-terminal domain serves as a redox activator of transcriptional factors (TFs) by regulating the DNA-binding activities of TFs. Because of the latter function, APE1 indirectly controls the expression of numerous genes from many different pathways. Consequently, APE1 participates in a wide range of processes, such as stress responses, cell survival, apoptosis, inflammation, angiogenesis, and many more [23,24].

Due to the fact that APE1 is a key protein engaged in various important mechanisms and/or processes, any abnormalities in its function can be deleterious for the organism. Similarly, any changes in *APE1* sequences may lead to serious defects and can even be lethal. For example, it was reported that the deletion of both alleles in mice promotes death in the early embryo stage [21].

Further analysis on *APE1^+/-^* mutant mice showed that diminution of *APE1* expression significantly increased spontaneous mutagenesis [25]. It was also presented that *APE1* knock-down in human fibroblasts led to an accumulation of DNA damage and apoptosis induction [26]. Moreover, aberrations in *APE1* sequences were described [27,28,29]. Over 50% of the substitutions of the single nucleotide lead to changes in the amino acid sequences [28], which may disturb proper cell functioning and increase the risk of diseases. Au and co-workers showed that blood lymphocytes treated with X-ray exhibited increased levels of DNA brakes and chromosome aberration. Their magnitudes were, in turn, dependent on specific polymorphisms variants within the sequences of DNA repair genes [30].

Yu and Hadi identified in silico over 80 missense mutations in *APE1* [31]. Although some of them are probably sequencing artifacts, other ones may be recognized as genetic risk factors. Because of the complexity and multi-factorial nature of many diseases, it is obvious that the latter, in most cases, are not determined exclusively by the specific genetic background. Environmental agents seem to play as important a role as genetic factors in the pathogenesis of numerous diseases. Therefore, individual mutations within *APE1* sequence should rather be considered as risk factors that in some cases (i.e., under specific environmental conditions) may lead to diseases [32]. The most widely studied single nucleotide polymorphisms (SNPs) that are associated with changes in the APE1 structure and/or activity as well as a wide range of diseases are listed in Table 1.

## 5. APE1 Role in Cell Cycle Regulation

It was shown that due to its specific structure [57], APE1 acts as a reductive activator that controls the status of the DNA binding domain of numerous transcription factors of TFs, including p53. Consequently APE1 affects their expression and activity [23,58]. With regard to p53, APE1 has been shown to co-localize with p53 in response to oxidative stress [59], which in turn promotes p53 tetramerization [60] and its binding to p21 promoter [61]. Thus, APE1 protects cells from damage accumulation. Zaky and co-workers proposed a model that describes a feedback between APE1 and p53 participating in determination of the cells’ fate, which is apoptosis vs. cell survival. After genotoxic stress, APE1 promotes p53 activity. In response to severe DNA damage, p53 downregulates *APE1* expression and protein levels [62] through ubiquitination [63], and thus inhibits DNA repair and promotes apoptosis [64]. Therefore, coordinated action of p53 and APE1 serves as a key regulator of genetic stability maintenance [65]. It was shown that cell lines with silenced p53 exhibit a slower removal of 8-oxoguanine (8-oxoG), the most common DNA lesion compared to wild-type cells. Sengupta et al. provided evidence that AP endonuclease plays a dual role in p21 regulation, with the latter suppressing cell proliferation and promoting cell cycle arrest. When p53 is presented in the cell, APE1 is stably bound to p53, promoting an increase of p21 expression. However, in p53-null cells, AP endonuclease represses p21 expression and promotes cell proliferation, which was observed in tumor tissue [61].

There are also reports suggesting that AP endonuclease regulates cell divisions in other ways. Vascotto et al. demonstrated that the silencing of *APE1* expression by using siRNA in HeLa cells disrupts the passage from S-G2/M phases to the subsequent G1 phase. Additionally, genome-wide analysis indicated 1126 genes that were differentially expressed after *APE1* knockdown. Among the proteins encoded by the upregulated genes, there were mostly cytoskeleton and microtubule components as well as proteins engaged in lipid metabolism and cell cycle arrest. In turn, protein products of downregulated genes mainly take part in protein biosynthesis, cell growth, and DNA repair. Interestingly, the silencing of *APE1* impaired the mitochondrial function, which was analyzed by membrane potential depolarization, suggesting that AP endonuclease may also regulate apoptosis through the intrinsic pathway [66].

## 6. APE1 Role in Telomere Stability Maintenance

Telomeres (TLs) are chromosomes’ end structures, the main function of which is to protect genome stability. TLs shorten during each round of replication, which is a natural phenomenon. However, accelerated telomere attrition was observed in many types of disease, such as bone marrow failure, fibrosis, metabolic disease, and cancer [67]. Interestingly, Taubel and coworkers analyzed the telomeres’ length in monocytes isolated from patients with heart failure. They demonstrated that after one year, the rate of telomere attrition exceeded 20% in patients [68], whereas in groups of healthy individuals, during ∼10 years of study, this value equaled 6% [69]. Similarly, a reduced TL length in MO was observed in patients with Alzheimer’s disease (AD) [70] and diabetes [71]. Furthermore, Ong and colleagues analyzed the telomere length, monocyte level, and cytokine concentration in serum of the elderly and young people. They provided evidence that the total monocyte count, especially non-classical MO, significantly increases with age. Furthermore, these monocytes had truncated telomeres and caused higher cytokine release into the serum. It was also proposed that the chronic inflammation observed in elderly people leads to age-related disease [72].

It is widely accepted that oxidative damage in telomeric sequences disturbs proper binding of telomeric repeat binding factor 1 (TRF1) and telomeric repeat binding factor 2 (TRF2) proteins to DNA, which create a critical complex for proper functioning of TL [73]. Moreover, interaction between TRF2 and BER protein was reported [74]. There is evidence demonstrating that APE1 participates in maintaining telomere length and stability. Our previous work reviewed the AP endonuclease role in TL protection [75]. The special role of APE1 in interaction with sheltering proteins, mainly TRF1, TRF2, and protection of telomeres protein 1 (POT1), was indicated [76]. Furthermore, APE1 is a critical factor for the stabilization of telomeres. It was found that a loss of APE1 leads to the depletion of TRF2 binding and genomic instability [77]. Burra and co-workers showed that APE1 N-terminal sequences are essential for controlling AP endonuclease enzymatic activity on telomeres’ structure [78]. Moreover, APE1 plays a pivotal role in cellular senescence and aging features and it is associated with interaction with sheltering proteins and telomere status [26].

The studies reported here, illustrating the protective effect of APE1 on telomeres, have been described in various cell lines, including fibroblasts, HeLa cells, and U2OS (human bone osteosarcoma epithelial cells). However, there is no evidence reporting the role of APE1 on TL biology in monocytes. Nevertheless, such a relationship may exist and is critical for maintaining the proper structure and genetic stability of telomeres in MOs. It would be interesting to address this issue in further studies.

## 7. The Extracellular Role of APE1 in Regulating ROS Levels and Immune Response

APE1 protein contributes significantly to the BER mechanism, as well as regulating transcriptional activities. Nevertheless, the efficiency of DNA damage repair strongly depends on the ROS level. Because AP endonuclease also has redox activity, this protein may control monocytes’ lifespan by increasing the MO antioxidant capacity. As it was mentioned above, MOs are particularly vulnerable to oxidative stress and are impaired in DNA repair mechanisms [15]. Therefore, the ROS level, which in turn results from the activity of both MO and other immune system cells (such as macrophages and granulocytes), is a crucial factor determining the monocyte count [79]. Interestingly, APE1 has an ability to reduce oxidative stress [80,81,82] through a redox-dependent mechanism [83]. Reduction of the oxidative stress in response to AP endonuclease activity was described in different cell lines [83,84,85], but the molecular details of the relationship between the intracellular reactive oxygen species level and AP endonuclease activity to reduce the ROS concentration in monocytes remain unclear. There is evidence that APE1 may physically interact with heat shock protein 70 (hsp 70) [86], which is involved in protein folding and protecting cells from oxidative stress. Additionally, APE1 may also regulate the ROS level in an indirect manner, through p53, which is able to change the ROS concentration by increasing the expression of genes encoding antioxidant enzymes, such as superoxide dismutase 2 (SOD2) and glutathione peroxidase 1 (GPx1) [87,88]. AP endonuclease increases the p53 level and the molecular details of this interaction are discussed above.

It is also believed that APE1 modulates the ROS level in an indirect manner through IL-6. Several independent research groups have shown that IL-6 decreases oxidative stress, stimulates the antioxidant response, and reduces apoptosis [89,90]. Xie et al. observed that overexpression of AP endonuclease was associated with a high secretary level of IL-6. Moreover, silencing of *APE1* expression by siRNA decreased the release of IL-6 [91]. Further studies also confirmed that there is a feedback between APE1 and IL-6. Park and coworkers provided evidence that AP endonuclease is released to plasma after lipopolysaccharide (LPS) injection in rats [92]. Moreover, the role of APE1 in host defense after pneumococcal meningitis was also described [93]. It was proposed that extracellular secretion of APE1 may be part of the immune response. Nath and colleagues demonstrated that AP endonuclease is released as extracellular vesicles by monocytes in response to inflammation. Then, APE1 is associated with the cell surface of monocytes, increases the expression and secretion of IL-6, and modulates the inflammatory response [94]. Therefore, it was shown that subsequent production of IL-6 stimulates APE1 translocation from the nucleus to the cytoplasm and additionally increases AP endonuclease secretion [91,94]. All those mechanisms protect cells and promote their survival after the increase of the ROS level. However, the balance between ROS production and neutralization is important in regulating some processes, such as cell maturation. An increasing ROS concentration and APE1’s role in stem cell differentiation and hematopoiesis was described [95,96]. Therefore, monocytes’ maturation to either dendritic cells or macrophages, which are able to repair DNA, may be part of an organism’s defense that protects against the accumulation of MOs with DNA damage. It is supposed that during monocytes’ differentiation, TNFα plays a crucial role. It was shown that stimulation of MO by TNFα promotes maturation to dendritic cells [97]. Interestingly, APE1 may modulate the TNFα level, which is described in the paragraph below. Direct regulation of monocyte maturation via APE1 has not been described. It can be speculated that AP endonuclease mediates this process; however, research is needed to illustrate the involvement of APE1 during monocyte differentiation.

## 8. The Protective Role of APE1 in Maintenance Genome Stability via TNFα-Regulated Pathways

The indirect role of APE1 in the regulation of genomic stability depends on the APE1-regulated inflammatory response, particularly through the control of tumor necrosis factor α (TNFα) production and/or its secretion [98]. Jedinak et al. reported that inhibition of APE1 redox function by E3330, which is an APE1 redox-specific inhibitor, exerted an anti-inflammatory effect in lypopolysaccharide (LPS)-stimulated macrophages (RAW264.7). The anti-inflammatory effect, upon APE1 inhibition, was executed by abolishment of nuclear factor kappa-light-chain-enhancer of activated B cells (NF-κB) function and probably other transcription factors (downstream of APE1), i.e., AP-1 and CREB, that in turn resulted in suppression of the secretion of inflammatory cytokines, including TNFα [98]. Given the fact that monocytes accumulate NF-κB in their cytoplasm to provide a rapid NF-κB response upon activation [99], one can assume that the abovementioned mechanism can also be found in these types of cells.

TNFα is a cytokine of pleiotropic functions, which is engaged in a wide range of biological processes, including inflammation [100,101]. Nevertheless, the role of TNFα is multi-faceted, given the fact that TNFα appears in two forms: soluble (sTNF) and membrane bound (mTNF). Moreover, through two different types of its receptors, that is TNFR1 and TNFR2, TNF triggers different signaling cascades [102,103]. Indeed, monocytes that are the main producers of TNFα, as only a few cells possess both TNFR1 and TNRF2 receptors. The former, which is found on most human cells, can be triggered by both sTNF and mTNF. In turn, TNRF2 is limited to immune cells and vascular endothelial cells and is triggered exclusively by mTNF [100,101,102,103]. As abovementioned, these two types of receptors are engaged in opposite TNF alpha signaling in monocytes. TNFR1 acts as a “proinflammatory” receptor, as autocrine binding of TNFα onto TNRF1 leads to increased production of proinflammatory cytokines. In turn, TNFR2 triggers mainly an anti-inflammatory pathway by upregulation of IL-10 [104].

Interestingly, there is a link between APE1 and TNFα. Park et al. documented an anti-inflammatory function of secreted APE1 in TNFα-stimulated human umbilical vein endothelial cells (HUVECs), a vascular inflammation model. The anti-inflammatory role of secreted APE1 was executed by APE1-mediated inhibition of TNFα binding to TNFR1. An equally important role in this signaling pathway was played by the acetylation status of APE1. The trichostatin A (TSA)-mediated hyperacetylation of APE1 (Ac-APE1) promoted its secretion but inhibited its redox activity. In contrast, the rapid Ac-APE1 deacetylation recovered the reducing activity of APE1. Such a conversion of Ac-APE1 to its native form inhibits inflammation through thiol-disulfide exchange-driven conformational change in the TNFR1 receptor [105].

Despite its effect on other cells and/or tissues, TNFα also affects monocytes themselves. Rushworth et al. [106] reported that TNF induced autocrine TNF expression, leading to prolonged activation of the nuclear factor erythroid 2-related factor 2 (Nrf2)-dependent antioxidant pathway in human monocytes. In particular, TNF mediated upregulation of Nrf2 and Nrf2-dependent genes, including NAD(P)H: quinone oxidoreductase 1 (NQO1), glutamyl cysteine ligase modulatory (GCLM), and glutathione S-transferase A (GSTA1). Interestingly, TNF stimulation did not induce expression of heme oxygenase-1 (HO-1) but even slightly decreased its mRNA level. Not surprisingly, Nrf2 induction by TNF was mediated by the production of reactive oxygen species, as Nrf2 is a key transcription factor engaged in the regulation of antioxidant and cytoprotective genes [107,108] also in monocytes [106], by binding to the antioxidant response element (ARE) located within promoter regions of its target genes. The biological significance of TNF-driven activation of the Nrf2-dependent antioxidant pathway seems to be aimed at protecting monocytes from TNF-induced overproduction of ROS and thus ROS-driven apoptosis [106]. Indeed, monocytes with silenced Nrf2 have been shown to decrease their viability upon TNF treatment, which in turn may result, as suggested by Rushworth et al. [106], also in uncontrolled inflammation’s progress. Therefore, it is an additional benefit of the Nrf2-dependent pathway’s activation. However, the question of why TNF decreases HO-1 expression, while simultaneously increasing the expression of other Nrf2-dependent genes, remains unanswered [106].

Similarly, the role of APE1 in regulating monocytic genome stability is not clear. On the one hand, as an endonuclease that operates in the impaired BER pathway (and in fact ends it, leaving DNA unrepaired), it significantly contributes to enhanced SSB and DSB levels in the monocytic DNA. In fact, monocytes are hypersensitive to ROS and ROS-driven apoptosis [15]. The biological significance of this pathway is probably related to regulation of the monocyte count, a reduced maturation of macrophages, the main ROS producer, and DCs, which altogether orchestrates a homeostatic regulation of the immune response. In this sense, the selective killing of monocytes is protective as it counteracts overactivation of the infection- and/or inflammation-induced immune response [15].

On the other hand, the role of APE1 in maintaining redox homeostasis and the protection of monocytes from oxidative damage to macromolecules, including DNA, seems to be in opposition to the first conclusion. Perhaps, APE1 is such a critical point in the maintenance of the subtle balance between monocytes’ apoptosis and their survival and/or, given the broader context, the sensor of different whole-body processes, including the immune response and oxidative stress signaling. Indeed, the direct link between Nrf2, oxidative stress signaling, and APE1 has been reported by Shan et al. [83]. In response to oxidative attack, APE1 activated Nrf2, which in turn induced expression of its downstream antioxidant genes. Moreover, APE1 has been shown to regulate steady-state production of cellular ROS [83]. Contrary to these findings are results obtained by Fishel et al. showing that inactivation of APE1 led to increased levels of its mRNA and protein [109].

## 9. Apurinic/Apyrimidinic Endonuclease 1 in Monocyte Cells as A Potential Target in Disease: Perspectives on Future Research

The results from various studies have confirmed that monocytes are particularly sensitive to ROS [15], but on the other hand, they could be a therapeutic target during radiation therapy. Effective radiation therapy combines two main mechanisms: Stopping the growth and proliferation of cancer cells and stimulating immune cells against the tumor [110]. The increase of the immune response after single low-dose radiation has potential clinical amplifications and were described in the reviews [111,112]. Falcke et al. showed that MOs were the most radio-resistant immune cells after an X-ray dose and more than 40% were still viable [113]. Additionally, it was suggested that low doses of X-ray have an immune-stimulatory response in monocytes, which was observed on an increase of pro-survival and proinflammatory signaling molecules [114]. In contrary, macrophages after radiation decreased the secretion of IL-1 [115] also confirming the view that MO may be an important target during radiation therapy, especially for their ability to regulate cytokine levels and the anti-tumor response [5].

Additionally, it was also proved that APE1 is secreted from monocytes after LPS treatment, and then, this extracellular APE1 localizes on the cell surface of the next primary monocyte cell membranes and modulates its inflammatory response [93]. All this suggests that through this mechanism, AP endonuclease may modulate local inflammation and mobilize the next immune cells [116,117]. However, this role of APE1 is particularly interesting in the context of the radiotherapy described above. It is interesting to check how the *APE1* expression and protein concentration in monocytes are changed after X-ray treatment. The role of APE1 in monocytes’ radioresistance and what pathways are promoted needs to be examined. The above evidence suggested that apurinic/apyrimidinic endonuclease 1 may be involved in monocyte-mediated immune cells’ modulation; however, future studies will be required to determine this role and the molecular mechanism in inflammatory diseases.

The potential role in the regulation of monocytes’ function and/or count through APE1 may also be exploited therapeutically in age-related diseases. Age-related changes in the immune system are often associated with a chronic inflammation process. Some of them, such as immunosenescence, are a consequence of cellular immune dysfunction, including deregulation of the inflammatory monocyte response [118]. As it was mentioned above, MOs are able to produce a wide range of cytokines and chemokines, induce local inflammation, and activate other immune cells. With age, it comes to TNF overproduction, which leads to chronic inflammation and impairs the immune system. Lowering levels of TNF may be an effective strategy in age-related diseases [119]. Puchta et al. showed that chronic exposure to TNF promotes premature egress from the bone marrow of MOs with increased inflammatory cytokine production, which, as a consequence, promotes prolonged inflammation and contributes to increasing the risk of age-related diseases [119]. However, TNFα was described as a crucial protein for monocyte survival. In an experiment conducted by Wofl and co-workers, a deficiency in TNF signaling increases cell death [120]. Interestingly, another research group presented results showing that TNF plays a dual role in the regulation of cell death and proliferation. They found that tumor necrosis factor at a low concentration decreases apoptosis; however, at higher concentrations, it has a pro-apoptotic function [121].

In this case, APE1’s role seems to be interesting to examine. Recently, a study characterized AP endnonuclease as a serological marker for vascular inflammation [122]. Moreover, Jin and co-workers observed that high concentrations of AP endonuclease strongly correlated with myocardial injury. Further analysis showed that circulating APE1 has anti-inflammatory effects and would protect against myocardial damage [45]. As it was mentioned above, AP endonuclease induces changes in TNF receptors that modulate inflammatory signals [105]. Therefore, through this ability, APE1 may also regulate both pro- and anti-apoptotic signals. However, future analyses are require to examine this role of AP endonuclease for modulate monocyte cell death pathways through TNF regulation.

The prolonged inflammation observed in age-related disease is also an effect of monocyte telomeres’ dysfunction. Telomere shortening and degradation were correlated with some diseases and described in a review [123]. Although some studies have revealed that the length of telomeres in monocytes is not correlated with AD [124], recent reports indicate that there is a statistically significant relationship [70]. Correlation between shortened telomeres in monocytes was also confirmed in heart failure [68] and in diabetes [71]. Therefore, telomeres’ degradation in leukocytes was also described in other pathologies, such as schizophrenia [125] and Parkinson’s [126]. Additionally, the protective role of APE1 in maintaining the proper telomere length was presented in our previous report [75]. However, from our data analysis, there are no direct studies linking the length of telomeres in monocytes with the modulating effect of APE1. This relationship may play an important role in the survival of monocytes and have a regulatory impact on controlling prolonged inflammation. Future analyses need to be performed to describe this role of APE1 in the biology of telomeres in monocyte cells.

Summarizing and for the sake of clarity, our review presents a potential role of APE1 in monocyte genetic stability modulation. AP endonuclease is well-know BER protein, however, monocytes’ repair pathways are impaired, which is why we present a perspective on future research. In our consideration, we focused mainly on the known roles of APE1, through which it can act as a kind of ‘backup system’, modulating the genetic stability in monocytes. Nevertheless, future studies will be needed to verify our reflections.

## 10. Conclusions

APE1 is a multifunction protein that participates in different pathways, such as DNA repair, apoptosis, maturation, redox homeostasis, the regulation of transcriptional factors’ activity, and many more. Regulating so many mechanisms, often opposites, makes this protein particularly attractive in research of its exact role in controlling the processes in cells. In monocytes, the DNA repair mechanisms are impaired; however, APE1 plays a critical role in those cells, also in other ways. A decrease of ROS concentrations and promotion of the immune response are the first steps for maintaining the genetic stability. Moreover, regulating the cell cycle and feedback with p53 protein, which regulates apoptosis in monocytes with DNA damage accumulation, is also important in controlling the final fate of cells. Nevertheless, it should be remembered that the regulation of mechanisms in a cell is multifaceted; therefore, one factor can coordinate many often opposite pathways. Our considerations seem to be far-reaching; however, literature studies suggest that there is a lack of data on the above-discussed effect of APE1 on the regulation of the genetic stability in monocytes. Indeed, such results will facilitate the understanding of the basics of many diseases and will be helpful when creating targeted therapy. In this report, we described the key role in regulating monocytes’ lifespan; however, the molecular details of AP endonuclease’s role in these cells requires further studies.

## Figures and Tables

**Table 1:** SNPs (single nucleotide polymorphisms) in the *APE1* (apurinic/apyrimidinic endonuclease 1) sequence, their effects on protein structure and/or activity, and their association with diseases.

Nucleotide (Common/Position on Chromosome/Variant)	Amino acid (Common/Position/Variant)	SNP	Effects on APE1 Protein	Disease
C/20456008/G	Q/51/H	rs1048945	Impairment of the endoribonuclease function of APE1 [33]	colorectal cancer [34], endometrial cancer [35]
A/20456045/G	I/64/V	rs2307486	Impairment of the endoribonuclease function of APE1 [33]	acute lymphoblastic leukemia [36]
T/20456046/C	I/64/T	rs61730854	Changes in APE1 structure that modulate protein binding facility to DNA [37]	N/A
T/20454990/G	N/A	rs1760944	Increased expression level of APE1 [38]	breast cancer cervical [39], cancer [40], osteosarcoma [41]
T/20,456,995/G	D/148/E	rs1130409	Structural destabilizing effects [42], diminished endoribonuclease activity [33], reduced phosphatase activity in the 3’ RNA [43]	colorectal cancer [44], lung cancer [45], breast cancer [42], bladder cancer [46], vitiligo [47,48], renal cell carcinoma [49], gastric cancer [50], Amyotrophic Lateral Sclerosis (ALS) [51], Polycystic ovary syndrome (PCOS) [52]
C/20457260/T	R/237/C	rs375526265	Structural aberration, defects in DNA binding, a decrease in endonuclease activity [53,54], impaired ability to associate with BER protein [55]	endometrial cancer [35,56]
G/20457272/A	G/241/R	rs33956927	Changes in nuclease activity on nucleosome substrates [53], diminished endoribonuclease activity [33]	N/A
A/20457399/G	D/283/G	rs1393126543	Reduced endonuclease activity and interaction with other BER proteins [55]	ALS [51]
C/20457482/T	P/311/S	rs1803120	Changes in APE1 structure that modulate protein binding facility with DNA [37], decreased cleavage activity above 45 °C [53]	N/A
